# Early prediction of ARDS caused by non-pulmonary sepsis based on machine learning algorithms of inflammatory indicators and blood gas parameters

**DOI:** 10.3389/fmed.2025.1722756

**Published:** 2025-12-10

**Authors:** Bo Deng, Baolin Jia, Shiping Wu, Xiaojuan Wu

**Affiliations:** 1 Department of General Practice, Suining Central Hospital, Suining, Sichuan, China; 2 Department of Stomatology, Suining Central Hospital, Suining, Sichuan, China; 3 Department of Respiratory Medicine, Suining Central Hospital, Suining, Sichuan, China

**Keywords:** acute respiratory distress syndrome (ARDS), blood gas parameters, inflammatory indicators, machine learning, non-pulmonary sepsis

## Abstract

**Objective:**

Acute respiratory distress syndrome (ARDS) is a common complication in patients with non-pulmonary sepsis. Early identification and prediction of the occurrence of ARDS in non-pulmonary sepsis patients are of vital importance for timely intervention and improving the prognosis of these patients.

**Materials and methods:**

482 patients were included in this study. The Recursive Feature Elimination (RFE) method was employed to identify the key variables related to the prognosis of sepsis. The selected variables were used to construct nine different machine learning prediction models. To evaluate the performance of the model, we employed the Receiver Operating Characteristic (ROC) Curve, calibration curve, and Decision Curve Analysis (DCA). The clinical significance of the model was further analyzed through Shapley Additive Explanations (SHAP) analysis.

**Results:**

Through the RFE method, the final selected 11 variables. In the training set and test set, the AUC of the LightGBM model was 0.954 (95% CI: 0.933–0.973) and 0.923 (95% CI: 0.864–0.967) respectively. In this study, the calibration curve of the LightGBM model was close to the diagonal, indicating that its probability predictions were relatively reliable. In the DCA curves, the LightGBM model consistently maintained the highest net gain within the threshold range of 0–0.4, indicating LightGBM has greater clinical practical value. Through SHAP analysis, it was found that the SOFA score, PaO2/FiO2 ratio, lactate level, creatinine, and SAPS II score were the five most important features in the model prediction.

**Conclusion:**

In this study, a machine learning model based on inflammatory indicators and blood gas parameters was successfully developed and validated to predict the risk of ARDS in patients with non-pulmonary sepsis.

## 1 Introduction

Sepsis is a life-threatening organ dysfunction caused by the dysregulation of the body’s response to infection, and it is a significant clinical challenge in the field of critical care medicine (1–3). Although pulmonary infection is the most common trigger of sepsis, non-pulmonary sepsis also has a high incidence and mortality rate, and its unique etiology, pathogenesis, and clinical characteristics deserve special attention (4, 5). Non-pulmonary sepsis refers to infections that originate in tissues or organs other than the lungs, including abdominal infections, genitourinary system infections, skin and soft tissue infections, bloodstream infections, and catheter-related infections, etc. (6, 7). Acute respiratory distress syndrome (ARDS) is a critical condition characterized by acute, diffuse lung inflammation and pulmonary edema, resulting in severe hypoxemia (8, 9). Although direct lung damage (such as pneumonia and aspiration) is the most common cause of ARDS, as an example of indirect lung injury, non-pulmonary sepsis is one of the most important causes of ARDS.

Although the medical standards are gradually improving, the treatment methods for ARDS mainly rely on supportive treatments. Therefore, ARDS remains the primary cause of high mortality rates in intensive care units (ICU). Previous studies have shown that the incidence of ARDS in ICU is approximately 10%, while the mortality rate is as high as over 40%. In response to this, developing effective models to predict the early occurrence of ARDS in the ICU and promptly implementing intervention measures is of great significance. Currently, there are studies that have attempted to predict ARDS caused by pulmonary sepsis (10, 11). Therefore, this research aims to construct a machine learning model using some inflammatory indicators and blood gas parameter indicators to predict ARDS caused by non-pulmonary sepsis at an early stage.

## 2 Materials and methods

### 2.1 Data sources

**Figure 1:**
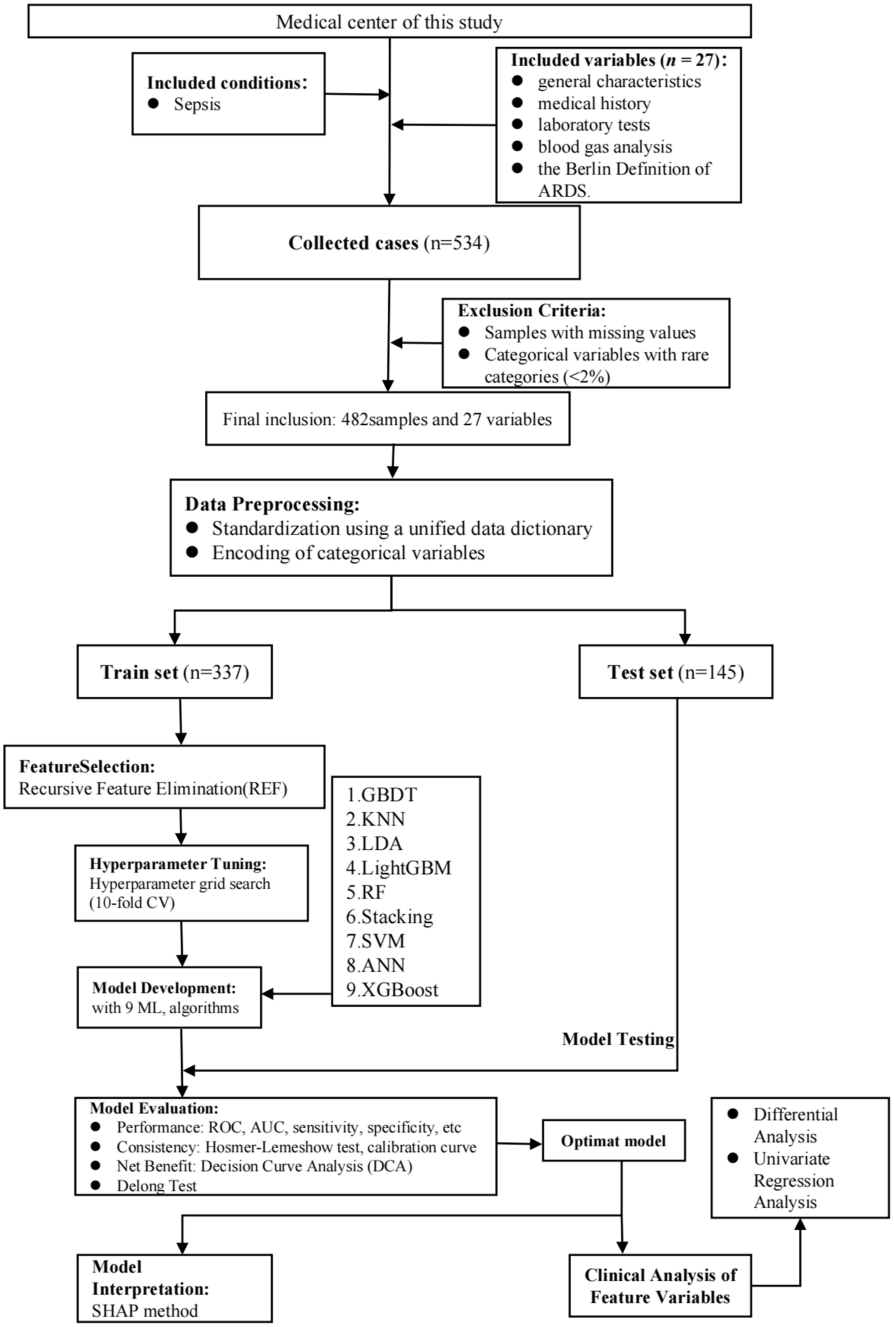
Flowchart of this study.

Figure 1 presents the overall flowchart of this study. The data for this study were collected from patients with sepsis admitted to the Intensive Care Unit of our medical center from 2021 to 2024. The data were extracted from the hospital’s electronic medical record (EMR) system, and all data were identifiable information. To ensure the ethical compliance of the study, this study has been approved by the hospital’s ethics committee, and all participants have signed informed consent forms. Additionally, the research methods strictly followed the relevant ethical requirements and regulations of the Helsinki Declaration.

### 2.2 Patient selection

According to the definition of Sepsis-3, sepsis is characterized by suspected infection combined with an acute increase of ≥ 2 points in the SOFA score. All the information of the participants was obtained from the EMR system of this hospital. On the basis of meeting the diagnostic criteria for sepsis, this study included patients aged over 18 years old and who did not develop ARDS during or within 2 days after admission. Exclusion criteria were: (1) Patients admitted to the ICU for less than 24 h; (2) Patients who developed ARDS during or within 2 days after admission, especially those caused by pulmonary factors. These patients may be unable to accurately assess the clinical outcomes related to sepsis due to the influence of their primary lung diseases or respiratory failure, and thus were excluded from the study; and (3) Samples with missing values and categorical variables with rare categories (<2%). This study only used the records of their first admission to the ICU. After screening according to the inclusion and exclusion criteria, a total of 482 patients with sepsis were included in this study.

### 2.3 Clinical variable collection

We extracted the following variables for the selected patients from the EMR system, including demographic data, age, gender, marital status, body mass index (BMI, kg/m2), and the patient’s previous medical history. We also recorded the vital signs and laboratory data within 48 h after admission to the ICU, including peripheral blood cell count (blood routine), blood gas parameters, and inflammatory indicators such as procalcitonin. The blood routine included tests such as hemoglobin levels, white blood cell count, neutrophils, and lymphocytes, which were obtained from peripheral blood samples taken during the patient’s initial assessment. If the patient underwent more than one laboratory test during hospitalization, only the initial test results were included in this study. The diagnosis of ARDS was in accordance with the Berlin criteria of the MIMIC-IV database. Berlin criteria: acute onset, PaO2/FiO2 ≤ 300 mm Hg, positive end-expiratory pressure (PEEP) ≥ 5 cm H2O on the first day of ICU admission, bilateral lung infiltration on chest X-ray, and no heart failure.

Based on these indicators, prognostic nutritional index (PNI), SII, SIRI, and AISI composite inflammatory indicators were derived. The composite indicators are defined as follows: PNI = serum albumin (g/L) + 5 × lymphocyte count (×109/L), SII = platelet count (×109/L) × neutrophil count (×109/L)/lymphocyte count, SIRI = neutrophil count × monocyte count (×109/L)/lymphocyte count, AISI = platelet count × monocyte count × platelet count/lymphocyte count. These indicators were used as auxiliary variables in the study to help further analyze the immune response, nutritional status, and clinical prognosis of patients with sepsis.

### 2.4 Statistical analysis

This study conducted a comprehensive statistical analysis of all patient data included. Descriptive statistics were first performed to calculate the frequencies and percentages of categorical variables, as well as the means, standard deviations, medians, and quartiles for numerical variables. For categorical variables, chi-square tests or Fisher’s exact tests were used to compare differences between groups, while independent sample *t*-tests or Mann–Whitney U tests were employed for numerical variables, depending on the distribution characteristics of the data. All descriptive statistics and related tests were conducted using SPSS software (version 25.0). When class imbalance was present, the synthetic minority oversampling technique (SMOTE) was applied to address the imbalance and improve the prediction of the minority outcome class. In the feature selection stage, we applied the Recursive Feature Elimination (RFE) method to identify key variables related to sepsis prognosis. RFE was performed using the RandomForestClassifier from the sklearn.ensemble module, which recursively eliminated features based on their importance in the model, ultimately selecting the most predictive variables. The specific parameters of the RandomForest base estimator used in the RFE process are provided in Supplementary File 3. These selected variables were then used to build nine different machine learning models: gradient boosting trees (GBDT), K nearest neighbors (KNN), linear discriminant analysis (LDA), LightGBM, random forest (Random Forest), stacking model (Stacking), support vector machine (SVM), artificial neural network (ANN), and XGBoost. Hyperparameter tuning for each model was performed using grid search with 10-fold cross-validation. The hyperparameter search space and the final chosen parameters for each model are detailed in Supplementary File 4. Model construction and training were carried out in the Python 3.10.4 environment using relevant libraries, including the library for conventional machine learning models, the library for XGBoost, and the library for the LightGBM model. For handling missing data, we excluded observations with missing values, primarily due to patients’ short hospital stays, which prevented certain assessments from being completed. This exclusion ensured that only complete data were used for modeling, thereby maintaining the quality and integrity of the analysis. While multiple imputation (using the function) is a common approach for handling missing data, we chose not to apply this method due to the study’s limited sample size. The decision to exclude missing data was made to reduce potential bias and ensure that the final dataset was as accurate and reliable as possible. Finally, based on the best prediction model, the clinical impact curve (CIC) was drawn to evaluate the application effect and potential of the model in actual clinical practice. All statistical analyses and the development of machine learning models were completed in the Python 3.10.4 environment and related libraries. All statistical tests were two-sided, and the significance level was set at *p* < 0.05.

## 3 Results

### 3.1 Demographic baseline characteristics

This study divided the samples into a training set (337 cases) and a test set (145 cases) according to the random seed 42 in a ratio of 7:3. Baseline feature analysis showed that there were no significant differences between the two groups in major indicators such as age (*p* = 0.554), bicarbonate (*p* = 0.564), lactate (*p* = 0.215), pH (*p* = 0.398), PaO₂ (*p* = 0.735), and hemoglobin (*p* = 0.130). Additionally, the incidence of acute respiratory distress syndrome (ARDS) did not show a significant difference between the test group (40.00%) and the training group (33.53%) (*p* = 0.173). However, the incidence of kidney disease was significantly higher in the training group (32.94% vs. 20.69%, *p* = 0.007). No significant differences were observed in demographic characteristics such as gender, BMI, and marital status between the two groups (*p* values were 0.258, 0.409, and 0.636, respectively). In summary, except for the difference in kidney disease, the training group and the test group were balanced in other demographic and clinical characteristics, and had good comparability (Table 1).

**Table 1:** Baseline characteristics of the study population across training and test cohorts.

Variables	Total (*n* = 482)	Test (*n* = 145)	Train (*n* = 337)	*P*
Age, M (Q₁, Q₃)	66.00 (55.25, 75.00)	67.00 (56.00, 75.00)	65.00 (55.00, 75.00)	0.554
HCO3-, M (Q₁, Q₃)	22.84 (19.64, 25.92)	22.90 (19.49, 25.69)	22.77 (19.73, 26.03)	0.564
Lactate, M (Q₁, Q₃)	1.51 (1.15, 2.02)	1.45 (1.12, 1.96)	1.55 (1.18, 2.05)	0.215
PH, M (Q₁, Q₃)	7.39 (7.36, 7.41)	7.39 (7.36, 7.41)	7.39 (7.36, 7.41)	0.398
PaO2, M (Q₁, Q₃)	87.87 (80.12, 95.11)	87.84 (80.26, 95.12)	87.89 (80.08, 95.09)	0.735
PaO2/FiO2, M (Q₁, Q₃)	335.60 (264.63, 405.93)	345.53 (270.64, 413.57)	334.71 (262.63, 398.89)	0.390
Hemoglobin, M (Q₁, Q₃)	11.79 (10.26, 13.19)	11.76 (10.19, 12.70)	11.81 (10.31, 13.39)	0.130
Creatinine, M (Q₁, Q₃)	1.00 (0.70, 1.30)	0.90 (0.70, 1.30)	1.00 (0.70, 1.30)	0.784
SOFA, M (Q₁, Q₃)	30.00 (24.00, 38.00)	29.00 (24.00, 38.00)	30.00 (24.00, 37.00)	0.622
SAPS II, M (Q₁, Q₃)	3.00 (2.00, 5.00)	3.00 (2.00, 5.00)	3.00 (1.00, 5.00)	0.671
PNI, M (Q₁, Q₃)	46.92 (42.61, 49.75)	46.65 (42.25, 49.45)	47.05 (42.65, 49.90)	0.300
SII, M (Q₁, Q₃)	1369.88 (893.82, 2058.61)	1429.88 (1049.79, 2192.12)	1355.43 (861.87, 2028.29)	0.250
SIRI, M (Q₁, Q₃)	5.12 (2.73, 9.69)	4.78 (2.77, 8.79)	5.36 (2.67, 9.80)	0.381
AISI, M (Q₁, Q₃)	983.10 (496.83, 1874.07)	893.65 (483.66, 1724.38)	1021.42 (511.68, 1983.47)	0.356
Procalcitonin, M (Q₁, Q₃)	0.05 (0.02, 0.21)	0.05 (0.02, 0.17)	0.05 (0.02, 0.22)	0.824
Result, *n* (%)				0.173
No ARDS	311 (64.52)	87 (60.00)	224 (66.47)	
ARDS	171 (35.48)	58 (40.00)	113 (33.53)	
Diabetes, *n* (%)				0.791
No	412 (85.48)	123 (84.83)	289 (85.76)	
Yes	70 (14.52)	22 (15.17)	48 (14.24)	
Renal disease, *n* (%)				**0.007**
No	341 (70.75)	115 (79.31)	226 (67.06)	
Yes	141 (29.25)	30 (20.69)	111 (32.94)	
Malignant cancer, *n* (%)				0.509
No	425 (88.17)	130 (89.66)	295 (87.54)	
Yes	57 (11.83)	15 (10.34)	42 (12.46)	
Liver disease, *n* (%)				0.434
No	341 (70.75)	99 (68.28)	242 (71.81)	
Yes	141 (29.25)	46 (31.72)	95 (28.19)	
Myocardial infarct, *n* (%)				0.233
No	347 (71.99)	99 (68.28)	248 (73.59)	
Yes	135 (28.01)	46 (31.72)	89 (26.41)	
Leukemia, *n* (%)				0.784
No	392 (81.33)	119 (82.07)	273 (81.01)	
Yes	90 (18.67)	26 (17.93)	64 (18.99)	
Septic shock, *n* (%)				0.189
No	294 (61.00)	82 (56.55)	212 (62.91)	
Yes	188 (39.00)	63 (43.45)	125 (37.09)	
Pancreatitis, *n* (%)				0.407
No	412 (85.48)	121 (83.45)	291 (86.35)	
Yes	70 (14.52)	24 (16.55)	46 (13.65)	
Gender, *n* (%)				0.258
Male	188 (39.00)	51 (35.17)	137 (40.65)	
Female	294 (61.00)	94 (64.83)	200 (59.35)	
BMI, kg/m^2^, *n* (%)				0.409
<18.5	166 (34.44)	42 (28.97)	124 (36.80)	
≥18.5 and <25	154 (31.95)	49 (33.79)	105 (31.16)	
≥25 and <32	151 (31.33)	50 (34.48)	101 (29.97)	
≥32	11 (2.28)	4 (2.76)	7 (2.08)	
Marital status, *n* (%)				0.636
Married	217 (45.02)	70 (48.28)	147 (43.62)	
Single	265 (54.98)	77 (51.72)	98 (56.38)	

Z, Mann–Whitney test; χ^2^, Chi-square test; M, Median; Q₁, 1st Quartile; Q₃, 3rd Quartile. Bold values indicate statistical significance (*p* < 0.05).

The baseline feature analysis shown in Table 2 revealed significant differences in multiple clinical indicators between the acute respiratory distress syndrome (ARDS) group and the non-ARDS group. There were no statistical differences in demographic characteristics such as age (*p* = 0.838), diabetes (*p* = 0.975), gender (*p* = 0.472), and marital status (*p* = 0.975) between the two groups. However, the ARDS group showed higher levels in multiple key clinical indicators. Specifically, the lactate concentration (1.74 mmol/L vs. 1.48 mmol/L, *p* = 0.003), creatinine level (1.10 mg/dL vs. 0.90 mg/dL, *p* < 0.001), SOFA score (38.00 vs. 28.00, *p* < 0.001), and SAPS II score (4.00 vs. 3.00, *p* = 0.006) of the ARDS group were significantly higher than those of the non-ARDS group. Conversely, the PaO₂/FiO₂ ratio (290.61 vs. 366.16, *p* < 0.001) and hemoglobin level (11.33 g/dL vs. 12.04 g/dL, *p* = 0.007) of the ARDS group were significantly lower than those of the non-ARDS group. These results collectively suggest that patients in the ARDS group had more severe physiological dysfunction and a heavier disease burden. Notably, there was no statistical difference in the incidence of kidney disease between the two groups (*p* = 0.127). In conclusion, although the two groups were similar in some demographic characteristics, the ARDS group showed significantly higher disease severity in key clinical indicators.

**Table 2:** Baseline characteristics of the study population across training cohorts.

Variables	Total (*n* = 337)	No ARDS (*n* = 224)	ARDS (*n* = 113)	*P*
Age, M (Q₁, Q₃)	65.00 (55.00, 75.00)	65.00 (55.00, 75.25)	66.00 (54.00, 74.00)	0.838
HCO3-, M (Q₁, Q₃)	22.77 (19.73, 26.03)	22.40 (19.75, 25.88)	23.55 (19.52, 26.32)	0.345
Lactate, M (Q₁, Q₃)	1.55 (1.18, 2.05)	1.48 (1.12, 1.96)	1.74 (1.33, 2.25)	**0.003**
Ph, M (Q₁, Q₃)	7.39 (7.36, 7.41)	7.39 (7.36, 7.41)	7.39 (7.36, 7.41)	0.527
PaO2, M (Q₁, Q₃)	87.89 (80.08, 95.09)	87.75 (80.01, 94.19)	88.73 (80.37, 95.68)	0.499
PaO2/FiO2, M (Q₁, Q₃)	334.71 (262.63, 398.89)	366.16 (285.13, 421.91)	290.61 (234.81, 345.00)	**<0.001**
Hemoglobin, M (Q₁, Q₃)	11.81 (10.31, 13.39)	12.04 (10.48, 13.60)	11.33 (9.89, 12.51)	**0.007**
Creatinine, M (Q₁, Q₃)	1.00 (0.70, 1.30)	0.90 (0.67, 1.20)	1.10 (0.90, 1.60)	**<0.001**
SOFA, M (Q₁, Q₃)	30.00 (24.00, 37.00)	28.00 (21.75, 33.00)	38.00 (29.00, 48.00)	**<0.001**
SAPS II, M (Q₁, Q₃)	3.00 (1.00, 5.00)	3.00 (1.00, 4.00)	4.00 (2.00, 5.00)	**0.006**
PNI, M (Q₁, Q₃)	47.05 (42.65, 49.90)	46.92 (42.65, 49.85)	47.35 (42.95, 50.15)	0.377
SII, M (Q₁, Q₃)	1355.43 (861.87, 2028.29)	1282.19 (848.31, 2014.69)	1417.14 (884.08, 2033.13)	0.379
SIRI, M (Q₁, Q₃)	5.36 (2.67, 9.80)	5.17 (2.62, 9.34)	5.93 (2.81, 10.54)	0.515
AISI, M (Q₁, Q₃)	1021.42 (511.68, 1983.47)	992.74 (513.67, 1869.72)	1054.14 (496.16, 2094.17)	0.666
Procalcitonin, M (Q₁, Q₃)	0.05 (0.02, 0.22)	0.05 (0.02, 0.18)	0.06 (0.02, 0.29)	0.299
Diabetes, *n* (%)				0.975
No	289 (85.76)	192 (85.71)	97 (85.84)	
Yes	48 (14.24)	32 (14.29)	16 (14.16)	
Renal disease, *n* (%)				0.127
No	226 (67.06)	144 (64.29)	82 (72.57)	
Yes	111 (32.94)	80 (35.71)	31 (27.43)	
Malignant cancer, *n* (%)				0.308
No	295 (87.54)	199 (88.84)	96 (84.96)	
Yes	42 (12.46)	25 (11.16)	17 (15.04)	
Liver disease, *n* (%)				0.323
No	242 (71.81)	157 (70.09)	85 (75.22)	
Yes	95 (28.19)	67 (29.91)	28 (24.78)	
Myocardial infarct, *n* (%)				0.277
No	248 (73.59)	169 (75.45)	79 (69.91)	
Yes	89 (26.41)	55 (24.55)	34 (30.09)	
Leukemia, *n* (%)				0.190
No	273 (81.01)	177 (79.02)	96 (84.96)	
Yes	64 (18.99)	47 (20.98)	17 (15.04)	
Septic shock, *n* (%)				0.146
No	212 (62.91)	147 (65.62)	65 (57.52)	
Yes	125 (37.09)	77 (34.38)	48 (42.48)	
Pancreatitis, *n* (%)				0.596
No	291 (86.35)	195 (87.05)	96 (84.96)	
Yes	46 (13.65)	29 (12.95)	17 (15.04)	
Gender, *n* (%)				0.472
No	137 (40.65)	88 (39.29)	49 (43.36)	
Yes	200 (59.35)	136 (60.71)	64 (56.64)	
BMI, kg/m^2^, *n* (%)				0.257
<18.5	124 (36.80)	78 (34.82)	46 (40.71)	
≥18.5 and <25	105 (31.16)	70 (31.25)	35 (30.97)	
≥25 and <32	101 (29.97)	73 (32.59)	28 (24.78)	
≥32	7 (2.08)	3 (1.34)	4 (3.54)	
Marital status, *n* (%)				0.975
Married	147 (43.62)	97 (43.30)	50 (44.25)	
Single	190 (56.38)	127 (56.70)	63 (55.55)	

Z, Mann–Whitney test; χ^2^, Chi-square test; −, Fisher exact; M, Median; Q₁, 1st Quartile; Q₃, 3rd Quartile. Bold values indicate statistical significance (*p* < 0.05).

### 3.2 Variable selection

In this study, we employed the RFE method for feature selection. To assess the importance of each feature, we selected the Random Forest as the base model. The Random Forest is capable of effectively measuring the contribution of features because it provides “importance” scores for each feature, which reflect the impact of the feature on the model performance.

During each iteration, the RFE algorithm recursively removes the least important features based on the importance scores of each feature in the Random Forest model. After each removal, the algorithm re-trains the Random Forest model and evaluates the performance of the new feature set. This process continues until the final optimal feature subset is reached. To ensure that the selected feature subset maintains good performance in future samples, we determined the stopping criteria based on the performance evaluation results of cross-validation. When the number of features decreased to the optimal level, the performance of the Random Forest model (such as accuracy, AUC value, etc.) remained stable or improved, thereby determining the stopping of feature selection.

**Figure 2:**
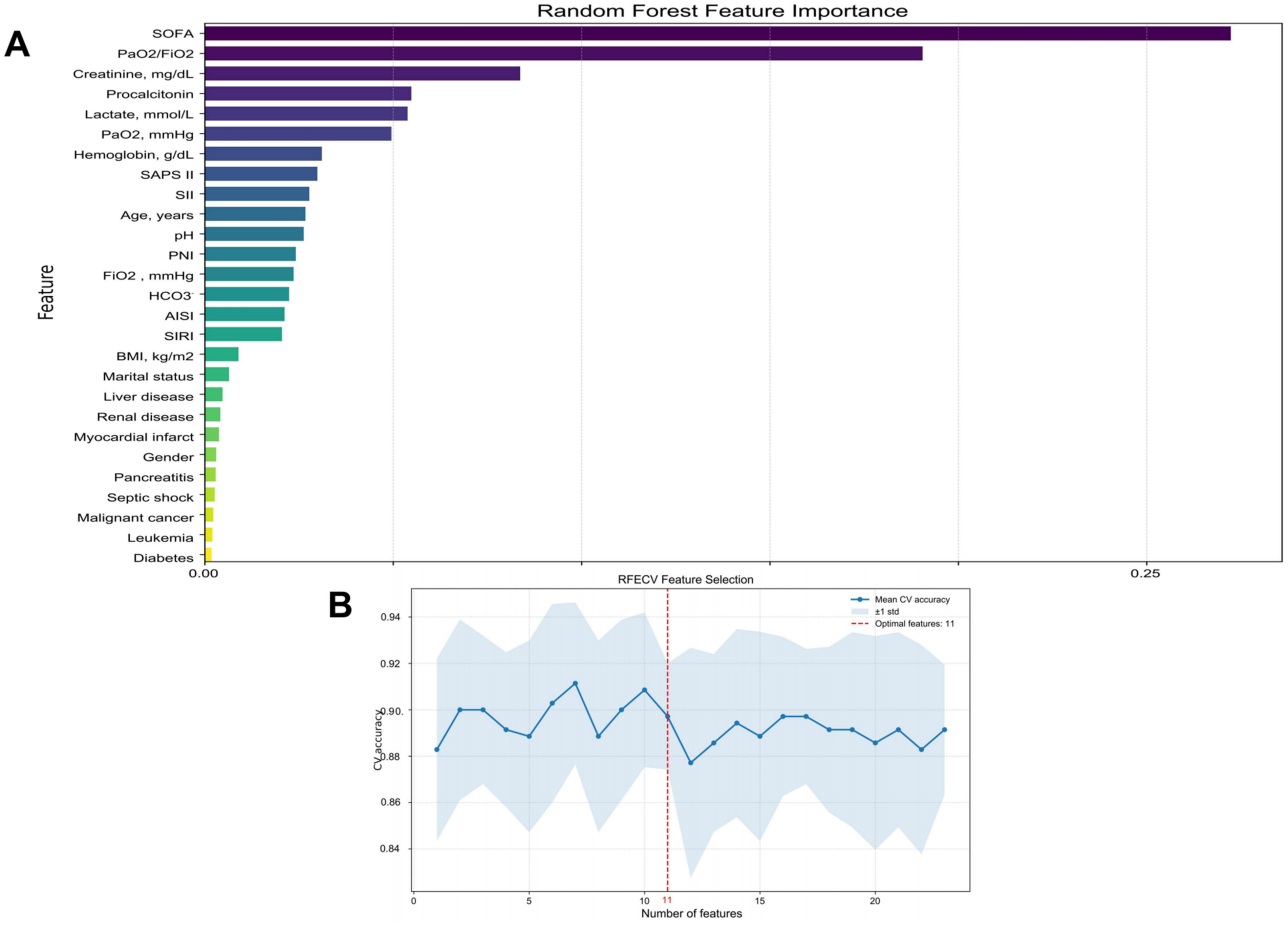
The ranking of feature importance in random forest **(A)**. Based on the random forest RFE (Recursive Feature Elimination) method, 11 variables were selected **(B)**.

Through the RFE method, the final 11 variables selected are HCO3-, PH, lactic acid, PaO2, PaO2/FiO2, Hemoglobin, Creatinine, SOFA, SAPS II, PNI, and Procalcitonin (Supplementary File 1). These features consistently demonstrated high importance scores in multiple iterations of the RFE algorithm, indicating their significant contribution in predicting and evaluating the clinical status and physiological indicators of patients with ARDS (Figure 2).

### 3.3 Performance of multiple machine learning models

**Figure 3:**
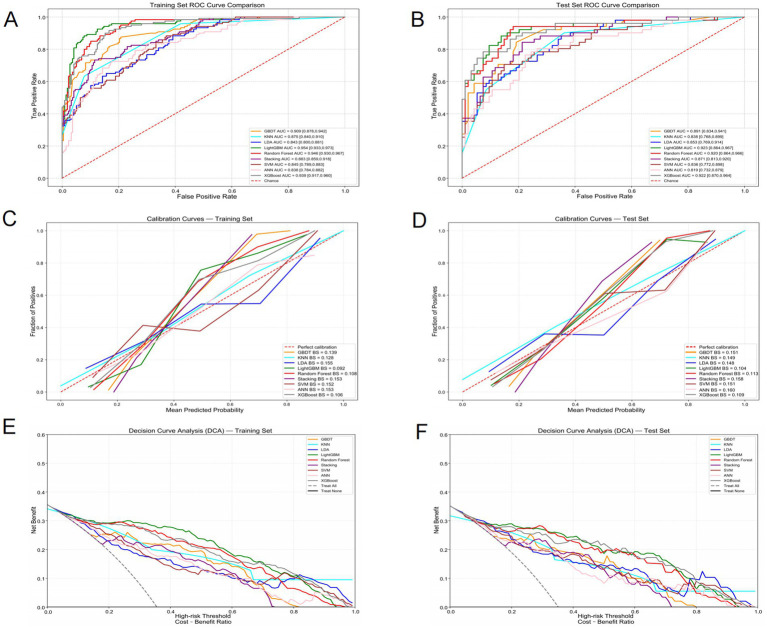
Comparison of ROC curves of different machine learning models in the training set **(A)** and the test set **(B)**. Comparison of Calibration curves of different machine learning models in the training set **(C)** and the test set **(D)**. Comparison of DCA curves of different machine learning models in the training set **(E)** and the test set **(F)**.

In this study, we screened multiple variables and constructed 9 different machine learning models. Each model was evaluated through 10-fold cross-validation to enhance the generalization ability of the models and avoid overfitting. To ensure the best performance of the models, we used grid search and random search methods to optimize the key hyperparameters. During the hyperparameter optimization process, we adjusted the important parameters of each model according to their characteristics, such as the penalty coefficient C of the Support Vector Machine (SVM), the number of decision trees in the Random Forest, and the number of neighbors in K-Nearest Neighbor (KNN). Finally, we selected the optimal hyperparameter combination and trained the model. On the training set, the AUC value of the LightGBM model was 0.954 (95% CI: 0.933–0.973), demonstrating the strongest predictive ability (Figure 3A). Although the AUC index can measure the predictive accuracy of the model, it cannot fully explain the effectiveness and clinical applicability of the model in practical applications. Therefore, on the basis of AUC evaluation, we further introduced calibration curves and decision curves, which can help us more comprehensively evaluate the actual predictive ability and application value of the model. First, the calibration curve compares the predicted probabilities of the model with the actual observed labels, revealing the degree of matching between the predicted probabilities and the true labels. In this study, the calibration curve of the LightGBM model was close to the diagonal, indicating that its probability prediction was relatively reliable. Additionally, we quantified the calibration performance of the model using the Brier score, which showed that the Brier score of LightGBM was the lowest (0.092), further confirming the excellent performance of this model in probability prediction and indicating that its output probabilities were most consistent with the true distribution, with strong calibration ability (Figure 3C). To further evaluate the practical application value of the model at different prediction thresholds, we drew decision curve analysis (DCA). DCA compares the net benefits at different prediction thresholds to measure the benefit of the model in practical applications. In this analysis, the LightGBM model maintained the highest net benefit within the threshold range of 0–0.4. Compared to other models and the baseline strategy, LightGBM had greater clinical practical value (Figure 3E). This indicates that in practical applications, choosing an appropriate prediction threshold (such as within the range of 0–0.4) can maximize the benefits of the model.

After the model evaluation on the training set, we further verified the generalization ability of the model using the test set. The performance of the LightGBM model on the test set was also excellent, with an AUC value of 0.923 (95% CI: 0.864–0.967), indicating that the model still maintained a high predictive ability on new data (Figure 3B). Additionally, the Brier score on the test set was 0.104. Although slightly higher than the result on the training set (0.092), it still indicated that the model maintained good performance in probability calibration (Figure 3D). For decision curve analysis (DCA), the performance of the LightGBM model on the test set was similar to that on the training set, still maintaining the highest net benefit within the threshold range of 0–0.4, further proving its consistency and practical application value across different datasets (Figure 3F).

Given the presence of class imbalance in the dataset, we further assessed model robustness under imbalanced conditions by applying SMOTE resampling to the training data. To evaluate robustness under class imbalance, the training data were resampled using SMOTE. After SMOTE adjustment, the LightGBM model maintained comparable performance, with stable AUCs (training cohort: 0.980, 95% CI: 0.957–0.995; test cohort: 0.957, 95% CI: 0.941–0.977) and good calibration. DCA also showed that the SMOTE-adjusted LightGBM model provided additional net clinical benefit across a broader range of thresholds (Supplementary File 2).

**Figure 4:**
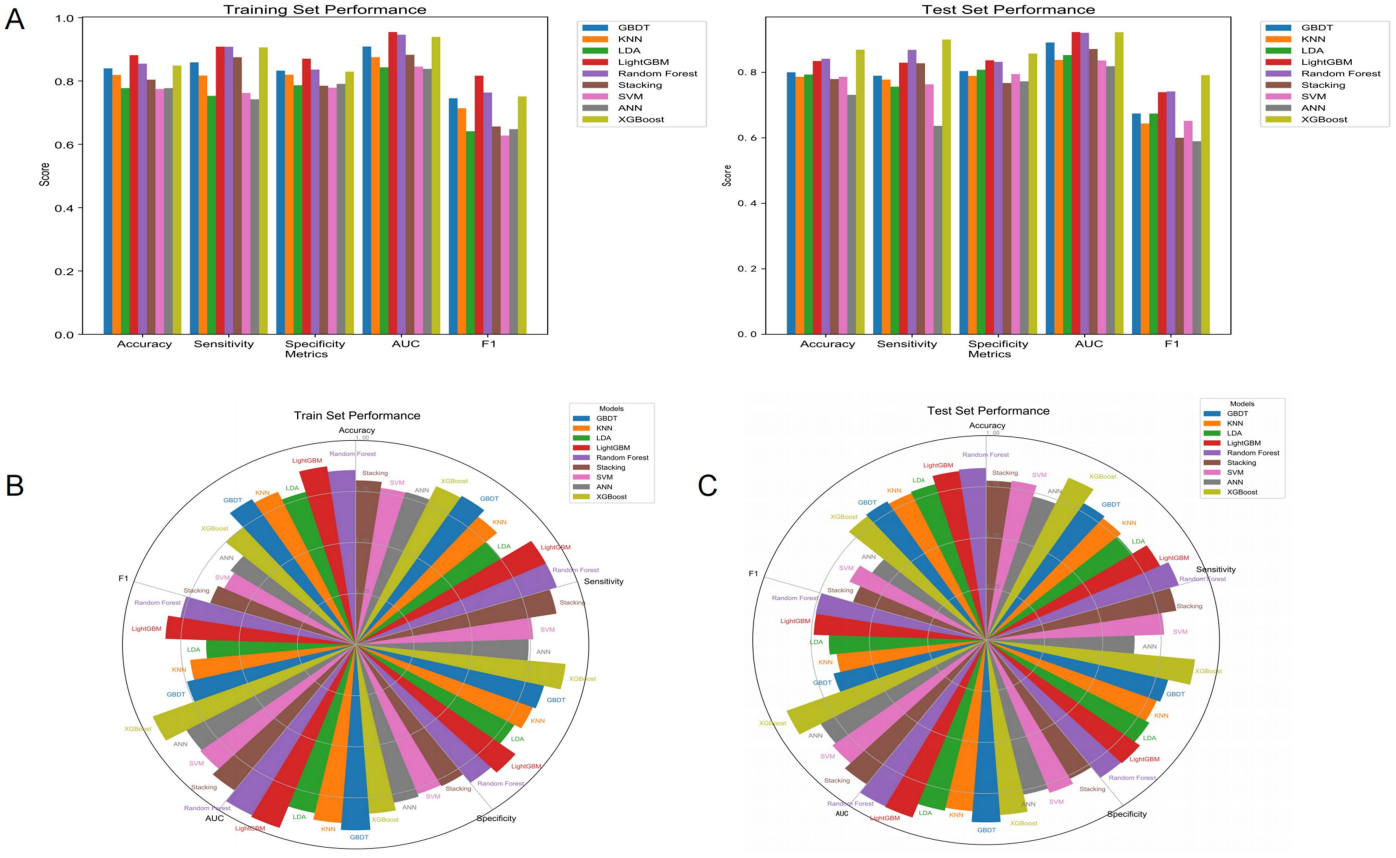
Performance metrics for various models on the training and test datasets. **(A)** Bar chart for training and test set performance, **(B)** Radar chart for training set performance metrics, **(C)** Radar chart for test set performance metrics. The performance metrics in all panels include accuracy, sensitivity, specificity, AUC, and F1.

To provide a more intuitive and comprehensive reflection of the classification performance of the model, we plotted the confusion matrices on the training set and the test set, and further calculated various indicators such as accuracy (Accuracy), sensitivity (Sensitivity/Recall), specificity (Specificity), precision (Precision), and F1 score (Figure 4; Table 3). Through the analysis of the confusion matrix, combined with AUC, Brier score, and DCA results, the stability and superiority of the LightGBM model on different datasets can be more clearly confirmed. To further verify the differences in prediction performance among different models, we used the DeLong test to compare the AUC of each model. Through the DeLong test, we were able to confirm that the LightGBM model has significantly superior predictive ability among all the evaluated machine learning models (Table 4).

**Table 3:** Performance of nine machine learning models in the training and test sets.

Data set	Model	Accuracy	Sensitivity	Specificity	C index	F1 Score
Train	GBDT	0.8398	0.8587	0.8327	0.9089	0.7453
	KNN	0.8190	0.8172	0.8197	0.8752	0.7136
	LDA	0.7774	0.7528	0.7863	0.8429	0.6411
	LightGBM	0.8813	0.9082	0.8703	0.9544	0.8165
	RF	0.8546	0.9080	0.8360	0.9460	0.7633
	Stacking	0.8042	0.8750	0.7849	0.8826	0.6563
	SVM	0.7745	0.7619	0.7787	0.8453	0.6275
	ANN	0.7774	0.7419	0.7910	0.8382	0.6479
	XGBoost	0.8487	0.9059	0.8294	0.9392	0.7512
Test	GBDT	0.8000	0.7895	0.8037	0.8910	0.6742
	KNN	0.7862	0.7778	0.7890	0.8377	0.6437
	LDA	0.7931	0.7561	0.8077	0.8525	0.6739
	LightGBM	0.8345	0.8293	0.8365	0.9228	0.7391
	RF	0.8414	0.8684	0.8318	0.9203	0.7416
	Stacking	0.7793	0.8276	0.7672	0.8711	0.6000
	SVM	0.7862	0.7632	0.7944	0.8360	0.6517
	ANN	0.7310	0.6364	0.7723	0.8185	0.5895
	XGBoost	0.8690	0.9000	0.8571	0.9222	0.7912

GBDT, Gradient Boosting Decision Tree; KNN, K-Nearest Neighbors; LDA, Linear Discriminant Analysis; LightGBM, Light Gradient Boosting Machine; RF, random forest; SVM, Support Vector Machine; ANN, Artificial Neural Network; XGBoost, Extreme Gradient Boosting.

**Table 4:** DeLong test for various models.

Model 1	Model 2	AUC1	AUC2	Difference	Z	*P*
GBDT	KNN	0.909	0.875	0.034	2.087	0.037
GBDT	LDA	0.909	0.843	0.066	4.086	0.000
GBDT	LightGBM	0.909	0.954	−0.046	−2.825	0.005
GBDT	Random Forest	0.909	0.946	−0.037	−2.306	0.021
GBDT	Stacking	0.909	0.883	0.026	1.630	0.103
GBDT	SVM	0.909	0.845	0.064	3.939	0.000
GBDT	ANN	0.909	0.838	0.071	4.377	0.000
GBDT	XGBoost	0.909	0.939	−0.030	−1.880	0.060
KNN	LDA	0.875	0.843	0.032	1.220	0.223
KNN	LightGBM	0.875	0.954	−0.079	−2.995	0.003
KNN	Random Forest	0.875	0.946	−0.071	−2.679	0.007
KNN	Stacking	0.875	0.883	−0.007	−0.279	0.780
KNN	SVM	0.875	0.845	0.030	1.130	0.259
KNN	ANN	0.875	0.838	0.037	1.397	0.162
KNN	XGBoost	0.875	0.939	−0.064	−2.419	0.016
LDA	LightGBM	0.843	0.954	−0.111	−5.322	0.000
LDA	Random Forest	0.843	0.946	−0.103	−4.922	0.000
LDA	Stacking	0.843	0.883	−0.040	−1.892	0.059
LDA	SVM	0.843	0.845	−0.002	−0.114	0.910
LDA	ANN	0.843	0.838	0.005	0.224	0.823
LDA	XGBoost	0.843	0.939	−0.096	−4.594	0.000
LightGBM	Random Forest	0.954	0.946	0.008	0.743	0.458
LightGBM	Stacking	0.954	0.883	0.072	6.373	0.000
LightGBM	SVM	0.954	0.845	0.109	9.677	0.000
LightGBM	ANN	0.954	0.838	0.116	10.304	0.000
LightGBM	XGBoost	0.954	0.939	0.015	1.352	0.176
Random Forest	Stacking	0.946	0.883	0.063	5.474	0.000
Random Forest	SVM	0.946	0.845	0.101	8.686	0.000
Random Forest	ANN	0.946	0.838	0.108	9.296	0.000
Random Forest	XGBoost	0.946	0.939	0.007	0.593	0.553
Stacking	SVM	0.883	0.845	0.037	2.059	0.039
Stacking	ANN	0.883	0.838	0.044	2.450	0.014
Stacking	XGBoost	0.883	0.939	−0.057	−3.129	0.002
SVM	ANN	0.845	0.838	0.007	0.342	0.732
SVM	XGBoost	0.845	0.939	−0.094	−4.541	0.000
ANN	XGBoost	0.838	0.939	−0.101	−4.533	0.000

Based on all the evaluation indicators, the LightGBM model performed the best on both the training set and the test set, and is the optimal model in this study.

### 3.4 Model explanation and SHAP analysis

**Figure 5:**
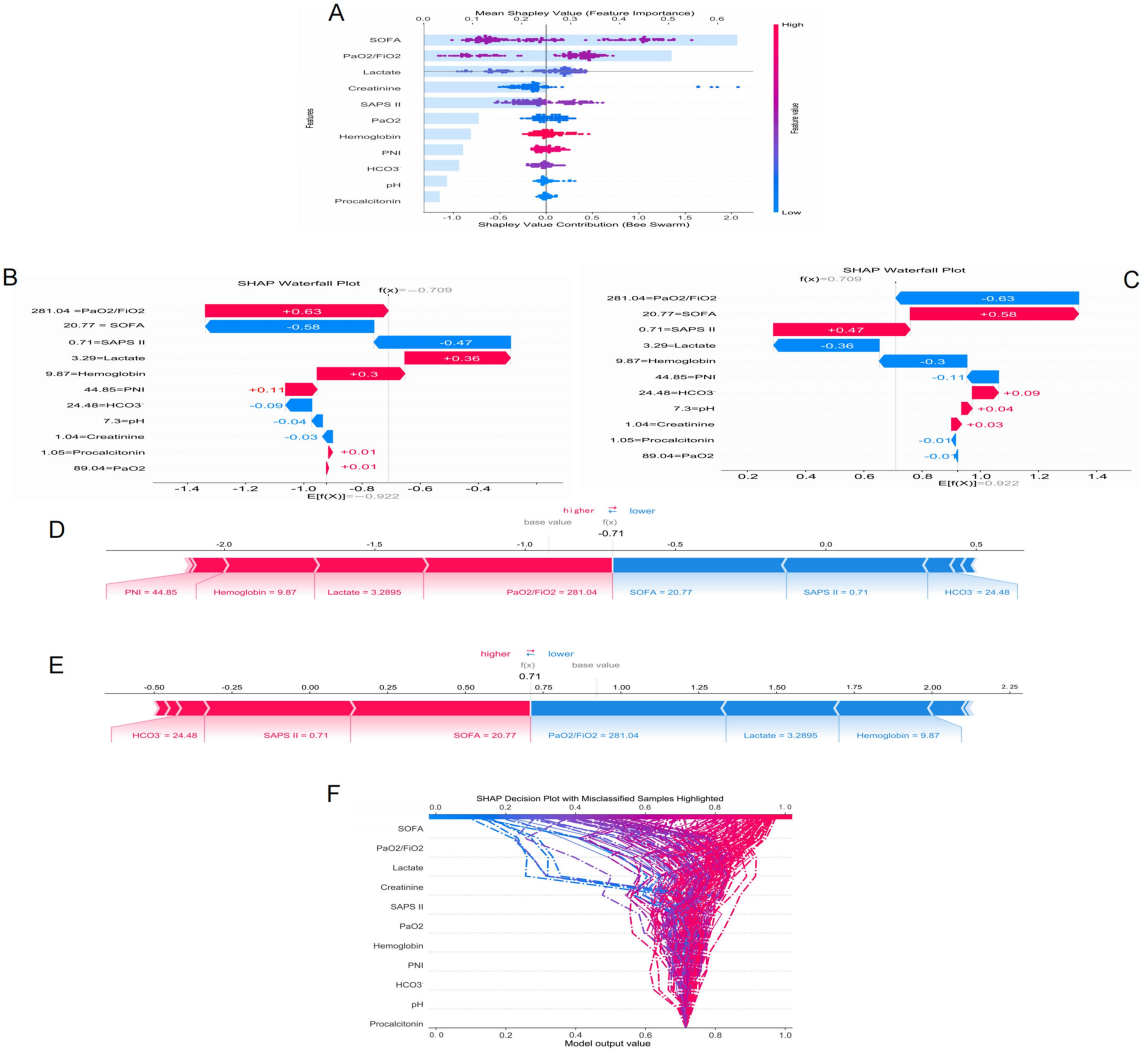
SHAP interprets the mode. **(A)** Attributes of characteristics in SHAP. Waterfall plots used to present the contribution of different characteristics to the model’s prediction output (SHAP values) for a particular patient **(B,C)**. Individual efforts by patients with ARDS **(D)** and without ARDS **(E)**. The SHAP value represents the predicted characteristics of an individual patient and the contribution of each characteristic to the predicted ARDS. SHAP analysis for decision-making **(F)**.

Figure 5 presents the SHAP analysis results of ARDS occurrence in sepsis, combining bar charts and beeswarm plots to quantify and visualize the contribution of each feature to the model’s prediction results. The SHAP value represents the degree of influence of each feature on the final prediction result under different feature combinations, with higher SHAP values indicating a greater impact of the feature on the model’s prediction. The bar chart in Figure 5A shows that the SOFA score, PaO2/FiO2 ratio, lactate level, creatinine, and SAPS II score are the five most important features in the model’s prediction, playing a decisive role in the prediction of ARDS. Additionally, Figure 5A also presents a beeswarm plot to visually display the distribution of SHAP values for each feature, showing the impact of different value ranges of each feature on the model’s prediction results. The beeswarm plot uses color coding (blue for lower feature values and red for higher feature values) to reveal the relationship between feature values and prediction results. Among all features, the SOFA score has the greatest impact on the model’s prediction, with higher values (indicated in pink) corresponding to higher SHAP values, indicating a strong influence on the prediction of ARDS. The PaO2/FiO2 ratio is also a key feature, with a wide range of SHAP values, and the higher the value (i.e., the higher the PaO2/FiO2), the higher the model’s prediction result. Other related features, such as lactate and creatinine, also have significant impacts on the prediction results, while features like procalcitonin have smaller contributions to the model’s prediction.

Figures 5B,C present waterfall plots, which show the contribution of different features to the model’s prediction output (SHAP value) for a specific patient. Each bar represents a feature, with blue bars indicating a negative contribution to the prediction result (i.e., reducing risk) and red bars indicating a positive contribution (i.e., increasing risk). Figures 5D,E present force plots, which illustrate how each feature jointly acts on the model’s baseline prediction value (base value) to form the final prediction value f(x). In these force plots, blue arrows indicate features that lower the prediction value, and red arrows indicate features that increase the prediction value. Figures 5B,C correspond to two different prediction results for the same sepsis patient regarding the occurrence of ARDS. When predicting the occurrence of ARDS, lactate (3.29) and PaO2/FiO2 (281.04) are the main risk-increasing factors, significantly increasing the model’s prediction value, suggesting a high risk of ARDS for this patient. Conversely, in the prediction of no ARDS occurrence for this patient, these features become risk-reducing factors, leading to a decrease in the model’s prediction value. Figures 5D,E present the prediction results for the same patient, with the analysis results being the same as above, further verifying the significant impact of lactate and PaO2/FiO2 on the prediction results.

To further understand the prediction mechanism of the model, Figure 5F presents a SHAP analysis decision plot. This decision plot visualizes the specific contribution of each feature to the model’s prediction result, with each curve representing a sample. The curve color changes from blue to red to indicate the change in feature values, with blue representing low risk and red representing high risk. The model output value (x-axis) reflects the predicted risk, with higher values indicating a greater risk. The figure also highlights samples with classification errors (indicated by dashed lines), helping to identify which features have a greater impact on the prediction results and which samples may have prediction errors. In this decision tree, features such as the SOFA score and the PaO2/FiO2 ratio contribute significantly to the model output, especially the SOFA score, which demonstrates strong predictive power. For misclassified samples, the contributions of features like SOFA and lactate vary greatly, revealing the model’s predictive uncertainty in certain extreme cases. These results provide important insights for further optimization and understanding of the model.

### 3.5 Clinical impact curves

**Figure 6:**
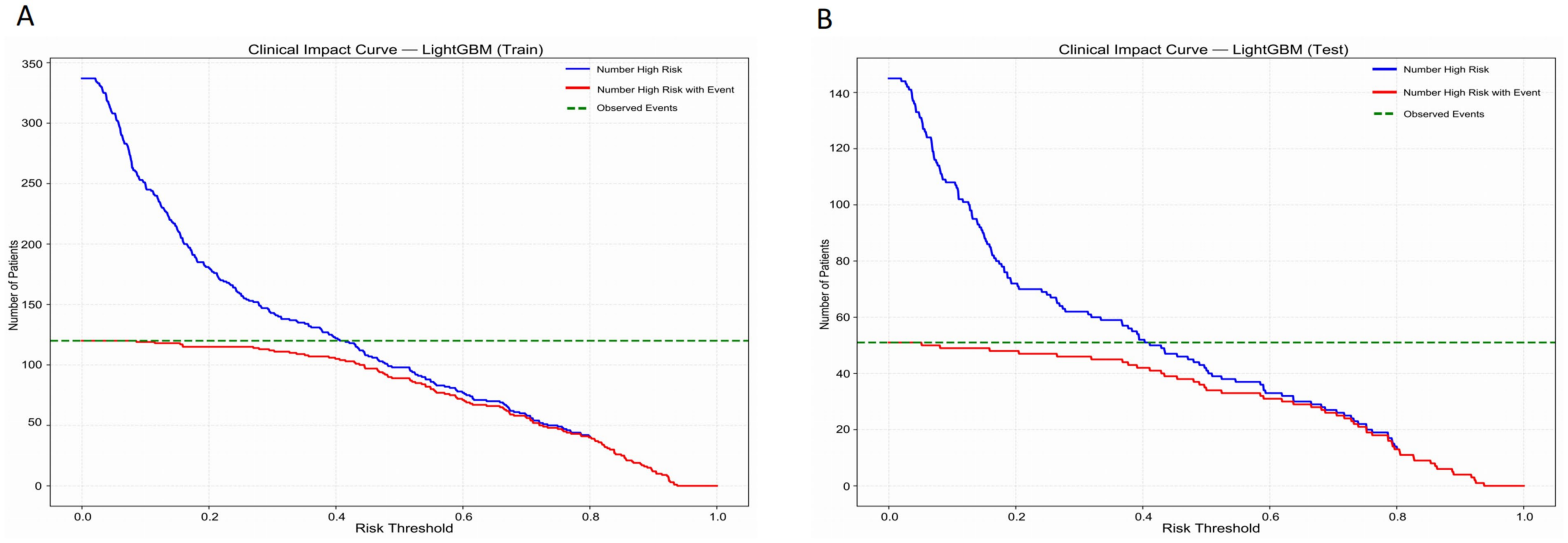
Clinical impact curves of the LightGBM model in the training set **(A)** and the test set **(B)**.

Figure 6 presents the Clinical Impact Curve of the LightGBM model, which is used to evaluate the clinical benefits of the model at different risk thresholds. Figures 6A,B respectively show the Clinical Impact Curves on the training set and the test set. In Figure 6A, as the risk threshold increases, the number of high-risk patients gradually decreases (the blue curve). The red curve represents the number of high-risk patients who actually experienced the event, which shows a different downward trend compared to the blue curve. The green dotted line represents the number of patients who actually experienced the event. To optimize the clinical application of the model, ideally, the red curve should be as close as possible to the blue curve, indicating that the proportion of events occurring in high-risk patients is higher. By adjusting the risk threshold, the model can effectively balance the proportion of identifying high-risk patients and the actual occurrence of events. Figure 6B shows a similar analysis on the test set, and the results are consistent with those of the training set, further verifying the generalization ability of the model. In the test set, although the number of high-risk patients and the number of actual events is relatively small, by adjusting the risk threshold, the model can still effectively identify high-risk patients and maintain good predictive performance. This analysis provides important evidence for clinical decision-making, helping to identify the high-risk patient groups with the most clinical significance at different thresholds.

## 4 Discussion

In this study, a machine learning model based on inflammatory indicators and blood gas parameters was successfully developed and validated to predict the risk of ARDS in patients with non-pulmonary sepsis. The RFE method was used to select variables such as HCO3-, pH, lactic acid, PaO2, PaO2/FiO2, Hemoglobin, Creatinine, SOFA, SAPS II, PNI, and Procalcitonin, which have significant contributions to predicting and evaluating the clinical status and physiological indicators of patients with ARDS. And multiple different machine learning models were constructed based on multiple variables. Eventually, it was found that the AUC of the LightGBM model in the training set and validation set was 0.954 (95% CI: 0.933–0.973) and 0.923 (95% CI: 0.864–0.967) respectively, demonstrating strong predictive ability. The calibration curve and DCA curve also indicate that LightGBM has good clinical application value. Based on all the evaluation indicators, the LightGBM model performed the best on both the training set and the test set, and it is the optimal model in this study. The clinical impact curve of LightGBM also provides an important basis for clinical decision-making, helping to identify the high-risk patient groups with the greatest clinical significance at different thresholds.

This study also utilized SHAP analysis to identify that the SOFA score, PaO2/FiO2 ratio, lactate level, creatinine, and SAPS II score were the five most important features in the model prediction. These key features played a decisive role in the prediction of ARDS. ARDS rarely exists independently. It is usually triggered by severe pulmonary or non-pulmonary diseases such as pneumonia, sepsis, and pancreatitis, and these causes themselves can lead to multiple organ dysfunction syndrome (MODS) (12, 13). The SOFA score is the “gold standard” tool for evaluating MODS. Moreover, numerous studies have confirmed that the baseline SOFA score at admission or when diagnosing ARDS is significantly correlated with in-hospital mortality. Therefore, the SOFA score plays an important role in predicting ARDS in both pulmonary and non-pulmonary sepsis. In clinical practice, for any patient with ARDS, calculating and tracking their SOFA score is a basic and crucial task. Non-pulmonary sepsis can lead to a systemic inflammatory response syndrome (14, 15). Inflammatory mediators, through circulation, can damage the endothelial cells of pulmonary vessels, resulting in pulmonary edema and affecting gas exchange, causing a sharp decline in the PaO₂/FiO₂ ratio. Therefore, the PaO₂/FiO₂ ratio is of great significance for monitoring patients with pulmonary sepsis or non-pulmonary sepsis (16). Timely detection of a decrease in this ratio and intervention is of great importance for preventing the occurrence of ARDS. Furthermore, the PaO₂/FiO₂ ratio is one of the most critical and fundamental objective indicators for assessing the severity of ARDS and predicting its prognosis (17–19). For patients with sepsis, an increase in lactate levels may be a precursor to ARDS. An increase in lactate levels is a sign of severe systemic hypoperfusion and cell damage, indicating a high risk of subsequent development of ARDS. Furthermore, the level of lactic acid is positively correlated with the severity of the disease. The higher the lactic acid level, the more severe the tissue hypoxia and cellular dysfunction, and the more critical the patient’s condition. Repeated monitoring of lactic acid levels and active intervention to reduce it are not only the core of anti-shock treatment, but also one of the key strategies for preventing and early response to ARDS (20–22). An increase in creatinine usually indicates acute kidney injury (AKI), and AKI and ARDS result from the parallel organ damage caused by the systemic inflammatory response and circulatory failure resulting from sepsis. They all point to a more dangerous clinical condition—multiple organ dysfunction syndrome (MODS). Therefore, an increase in creatinine levels is a warning sign of ARDS. The coexistence of these two conditions often indicates an extremely critical condition with a very poor prognosis (23–25). SAPS II is not a direct predictor of ARDS but rather a powerful integrated indicator of severity. It quantifies the underlying risk and overall background of patients developing ARDS. A high SAPS II score indicates a more severe systemic inflammatory response and poorer physiological reserve. As the main target organ of sepsis, the lungs are often subjected to more severe damage, thus the probability of developing ARDS is significantly higher. In clinical practice, SAPS II should be regarded as an efficient screening tool for quickly identifying patients with sepsis who require close monitoring to prevent the occurrence and development of ARDS (26–28).

Sepsis is prone to cause ARDS, and the mortality rate of patients is relatively high. Therefore, determining whether a sepsis patient will develop ARDS can provide assistance in predicting the prognosis of the patient (29). However, non-pulmonary sepsis is often overlooked by people. But non-pulmonary severe infections can lead to a systemic inflammatory response, thereby causing ARDS. Early prediction of ARDS in such patients can seize the treatment window, and it can improve prognosis and reduce mortality of patients (30). In this study, a machine learning model was constructed using some easily obtainable inflammatory indicators and blood gas analysis parameters from clinical practice, enabling the early detection of patients with non-pulmonary sepsis who may develop ARDS. This model is highly significant. Once these patients are identified, we can intervene promptly to prevent them from developing severe complications, thereby saving their lives. Furthermore, the variables involved in this study are readily available and have low costs, making them suitable for use in various medical centers at all levels. This demonstrates the practicality of the research results.

Although the machine learning model constructed in this study has a good predictive ability, there are still some limitations in this research. Firstly, the data for this study were obtained from a single-center, which may lead to selection bias. Furthermore, the conclusion of this study still lacks external data validation. Only internal validation cannot prove the general applicability of the model, and thus it cannot be further generalized. In the future, multi-center patient data are needed to reduce the influence of selection bias on the conclusion and to conduct external verification. This study is retrospective in nature, which may lead to some potential confounding variables interfering with the results. Future forward-looking research will enhance the feasibility of the conclusions drawn from this study. Then, although the AUC value of the model constructed in this study is high and the prediction performance is good, this might also indicate overfitting to the specific patterns in the data of this study, suggesting a risk of overfitting. This phenomenon may limit the model’s generalizability in independent datasets or other patient populations. Finally, the complexity of machine learning limits its ability for wide application. Better methods are needed to construct simple and practical models in the future. Moreover, future research should explore integrating more biomarkers to build models to improve the accuracy and reliability of predictions.

## 5 Conclusion

In this study, a machine learning model based on inflammatory indicators and blood gas parameters was successfully developed and validated to predict the risk of ARDS in patients with non-pulmonary sepsis. The research results indicate that the LightGBM model demonstrates strong predictive ability in identifying patients with non-pulmonary sepsis who develop ARDS, providing valuable contributions to personalized management strategies and early intervention.

## Data Availability

The original contributions presented in the study are included in the article/Supplementary material, further inquiries can be directed to the corresponding authors.

## Supplementary material

Supplementary File 2 ROC curve comparison, calibration curves, and decision curve analysis (DCA) for training and test sets. Panel **A** shows the ROC curve for the training set, Panel **B** shows the ROC curve for the test set, Panel **C** displays the calibration curve for the training set, Panel **D** shows the calibration curve for the test set, Panel **E** presents the decision curve analysis (DCA) for the training set, and Panel F shows the decision curve analysis (DCA) for the test set.
