# Supplementary figures and images for: Early prediction of ARDS caused by non-pulmonary sepsis based on machine learning algorithms of inflammatory indicators and blood gas parameters
Source: Front Med (Lausanne). 2025 Dec 10;12:1722756. doi: 10.3389/fmed.2025.1722756 (PMC12727968; doi:10.3389/fmed.2025.1722756)

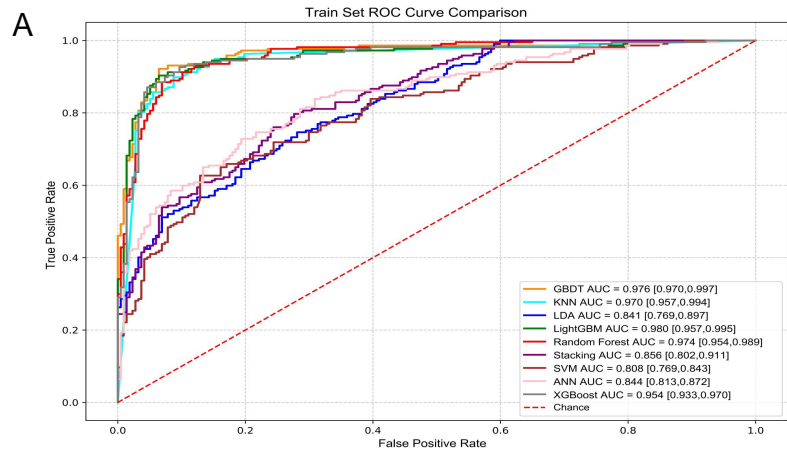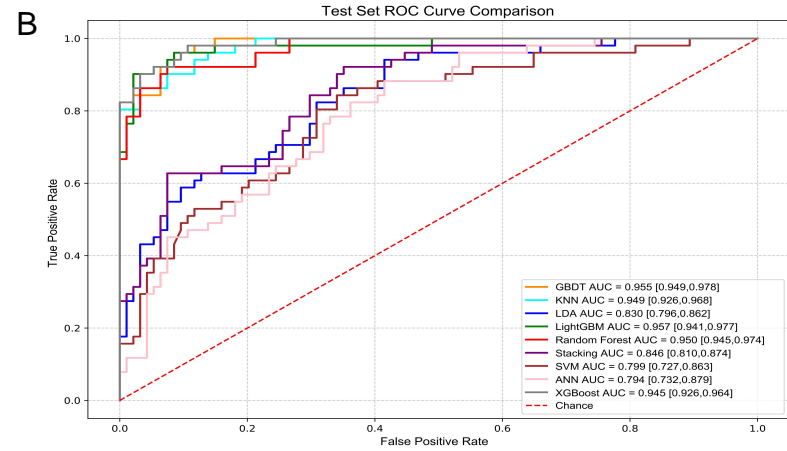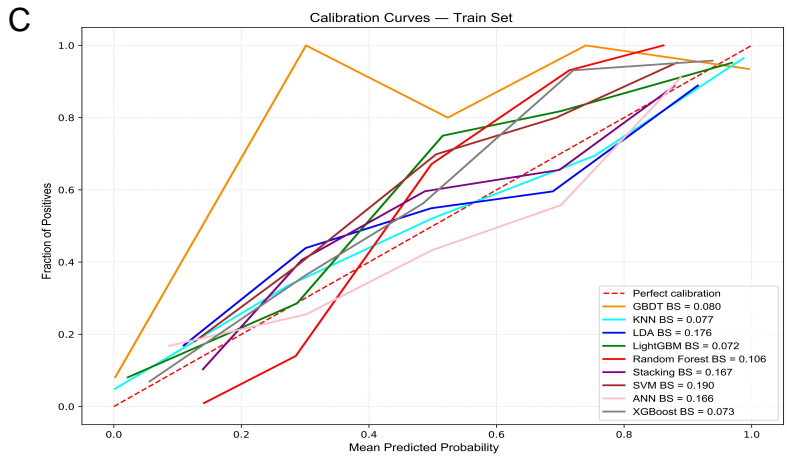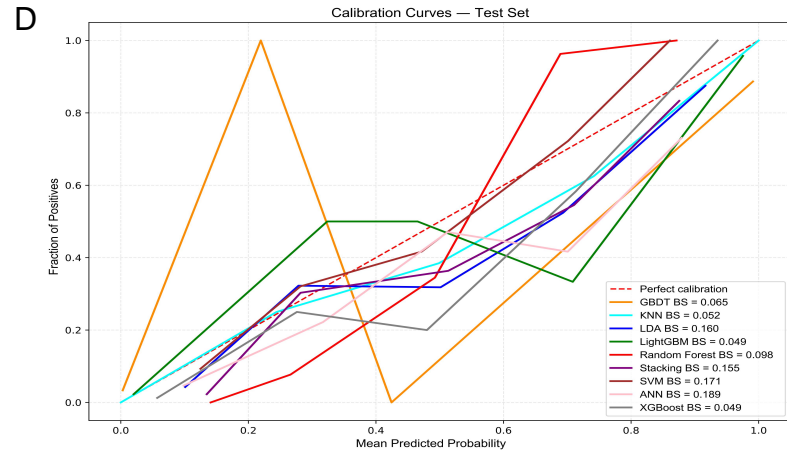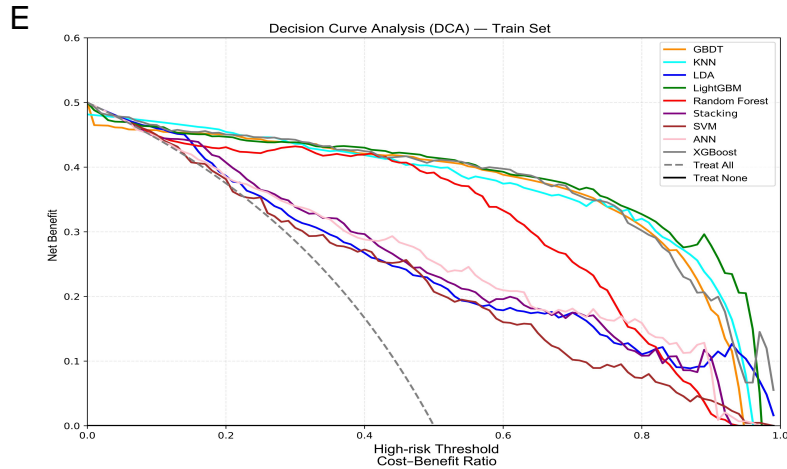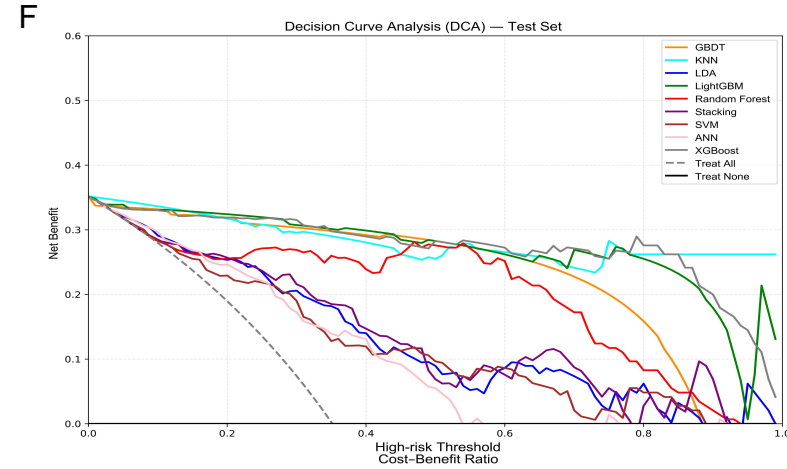

Supplement: Supplementary File 2 — ROC curve comparison, calibration curves, and decision curve analysis (DCA) for training and test sets. Panel A shows the ROC curve for the training set, Panel B shows the ROC curve for the test set, Panel C displays the calibration curve for the training set, Panel D shows the calibration curve for the test set, Panel E presents the decision curve analysis (DCA) for the training set, and Panel F shows the decision curve analysis (DCA) for the test set. [file Supplementary_file_2.pdf]
