# Supplementary material for: Early prediction of ARDS caused by non-pulmonary sepsis based on machine learning algorithms of inflammatory indicators and blood gas parameters
Source: Front Med (Lausanne). 2025 Dec 10;12:1722756. doi: 10.3389/fmed.2025.1722756 (PMC12727968; doi:10.3389/fmed.2025.1722756)
Supplement: Supplementary file 2 [file Supplementary_file_1.docx]

**Supplementary File 1. Comparison before and after including the variables.**

| **All Variables** | **Inclusion in final model** |
| --- | --- |
| Age |  |
| HCO3- | Yes |
| Lactate | Yes |
| PH | Yes |
| PaO2 | Yes |
| PaO2/FiO2 | Yes |
| Hemoglobin | Yes |
| Creatinine | Yes |
| SOFA | Yes |
| SAPS II | Yes |
| PNI | Yes |
| SII |  |
| SIRI |  |
| AISI |  |
| Procalcitonin | Yes |
| Diabetes |  |
| Renal disease |  |
| Malignant cance |  |
| Liver disease |  |
| Myocardial infarct |  |
| Leukemia |  |
| Septic shock |  |
| Pancreatitis |  |
| Gender |  |
| BMI |  |
| Marital status |  |
| Result |  |
