# Supplementary material for: Early prediction of ARDS caused by non-pulmonary sepsis based on machine learning algorithms of inflammatory indicators and blood gas parameters
Source: Front Med (Lausanne). 2025 Dec 10;12:1722756. doi: 10.3389/fmed.2025.1722756 (PMC12727968; doi:10.3389/fmed.2025.1722756)
Supplement: Supplementary file 3 [file Supplementary_file_3.docx]

**Supplementary File 3: Parameters of RFE**

# 1.载入相关库

from sklearn.feature_selection import RFECV

from sklearn.ensemble import RandomForestClassifier

from sklearn.model_selection import StratifiedKFold

# 2. 设置RFECV参数

rfecv = RFECV(

    estimator=rf,  # 基础模型

    step=1,  # 每次迭代移除的特征数

    cv=StratifiedKFold(10),  # 分层10折交叉验证

    scoring='accuracy',  # 评估指标

    min_features_to_select=3  # 最少保留的特征数

)

# 3. 执行特征选择

rfecv.fit(X_train, y_train)

# 4. 输出结果

print("最优特征数: %d" % rfecv.n_features_)

print("被选中的特征:")

selected_features = X_train.columns[rfecv.support_]

print(selected_features.tolist())

# 5. 绘制交叉验证结果

plt.figure(figsize=(10, 6))

plt.plot(range(1, len(rfecv.cv_results_['mean_test_score']) + 1),

         rfecv.cv_results_['mean_test_score'])

plt.fill_between(range(1, len(rfecv.cv_results_['mean_test_score']) + 1),

                 rfecv.cv_results_['mean_test_score'] - rfecv.cv_results_['std_test_score'],

                 rfecv.cv_results_['mean_test_score'] + rfecv.cv_results_['std_test_score'],

                 alpha=0.1)

# 添加最优特征数量标记线

plt.axvline(x=rfecv.n_features_, color='red', linestyle='--',

            label=f'最优特征数: {rfecv.n_features_}')

plt.xlabel("特征数量")

plt.ylabel("交叉验证准确率")

plt.title("RFECV特征选择结果")

plt.grid(True)

plt.tight_layout()

plt.show()
