# Supplementary material for: Early prediction of ARDS caused by non-pulmonary sepsis based on machine learning algorithms of inflammatory indicators and blood gas parameters
Source: Front Med (Lausanne). 2025 Dec 10;12:1722756. doi: 10.3389/fmed.2025.1722756 (PMC12727968; doi:10.3389/fmed.2025.1722756)
Supplement: Supplementary file 4 [file Supplementary_file_4.docx]

**Supplementary File 4: Parameters of the nine machine learning algorithms**

**1.KNN**

# 创建KNN分类器对象

knn = KNeighborsClassifier()

# 定义要搜索的超参数网格

param_grid = {

    "n_neighbors": np.arange(1, 11),

    "weights": ["uniform", "distance"],

    "p": [1, 2]

}

# 创建网格搜索对象

grid_search = GridSearchCV(knn, param_grid, cv=10)

# 在训练集上进行网格搜索

grid_search.fit(X_train, y_train)

#获取最佳参数

best_params=grid_search.best_params_

print("网格搜索的最佳参数：",best_params)

best_model = grid_search.best_estimator_

y_pred_train = best_model.predict(X_train)

y_pred_test=best_model.predict(X_test)

[accuracy_train,sensitivity_train,specificity_train,positive_predictive_values_train,negative_predictive_value_train,f1_train,kappa_train]=calculate_metrics_two(y_pred_train,y_train)

[accuracy_test,sensitivity_test,specificity_test,positive_predictive_values_test,negative_predictive_value_test,f1_test,kappa_test]=calculate_metrics_two(y_pred_test,y_test)

**2.RandomForest**

# 定义超参数的分布

param_distributions = {

    "n_estimators": np.arange(10, 201, 10),

    "max_depth": [None] + list(np.arange(3, 14)),

    "min_samples_split": np.arange(2, 11),

    "min_samples_leaf": np.arange(1, 6),

    "max_features": ["auto", "sqrt", "log2", None] + list(np.arange(1, X_train.shape[1] + 1))

}

# 创建随机搜索对象

random_search = RandomizedSearchCV(rf, param_distributions, n_iter=50, cv=10, random_state=42)

random_search.fit(X_train,y_train)

#获取最佳参数

best_params=random_search.best_params_

print("随机搜索的最佳参数：",best_params)

best_model = random_search.best_estimator_

y_pred_train = best_model.predict(X_train)

y_pred_test=best_model.predict(X_test)

[accuracy_train,sensitivity_train,specificity_train,positive_predictive_values_train,negative_predictive_value_train,f1_train,kappa_train]=calculate_metrics_two(y_pred_train,y_train)

[accuracy_test,sensitivity_test,specificity_test,positive_predictive_values_test,negative_predictive_value_test,f1_test,kappa_test]=calculate_metrics_two(y_pred_test,y_test)

**3.stacking**

# 定义基础分类器

base_classifiers = [

    ('decision_tree', DecisionTreeClassifier(random_state=42)),

    ('lda', LinearDiscriminantAnalysis()),

    ('svm', SVC(probability=True, random_state=42))

]

# 定义元分类器

meta_classifier = LogisticRegression()

# 创建StackingClassifier

stacking_clf = StackingClassifier(estimators=base_classifiers, final_estimator=meta_classifier)

# 定义超参数网格

param_distributions = {

    'decision_tree__max_depth': [3, 5, 7],              # 决策树的最大深度

    'svm__C': [0.1, 1, 10],                             # SVM的正则化参数

    'svm__kernel': ['linear', 'rbf'],                   # SVM的核函数

    'final_estimator__C': [0.1, 1, 10]                  # 元分类器Logistic Regression的正则化参数

}

# 创建随机搜索对象

random_search = RandomizedSearchCV(stacking_clf, param_distributions, n_iter=3, cv=10, random_state=42,verbose=3)

random_search.fit(X_train,y_train)

#获取最佳参数

best_params=random_search.best_params_

print("网格搜索的最佳参数：",best_params)

best_model = random_search.best_estimator_

y_pred_train = best_model.predict(X_train)

y_pred_test=best_model.predict(X_test)

[accuracy_train,sensitivity_train,specificity_train,positive_predictive_values_train,negative_predictive_value_train,f1_train,kappa_train]=calculate_metrics_two(y_pred_train,y_train)

[accuracy_test,sensitivity_test,specificity_test,positive_predictive_values_test,negative_predictive_value_test,f1_test,kappa_test]=calculate_metrics_two(y_pred_test,y_test)

**4.SVM**

# 创建SVM分类器对象

svc = SVC(random_state=42)

param_distributions = {

    'C': [0.001,0.01,0.1,1,10,100],  # 正则化参数

    'gamma': [0.001,0.01,0.1,1,10,100],  # 核函数参数

    'kernel': ['linear', 'rbf', 'sigmoid'],  # 核函数类型    #注poly核函数跑的话特别消耗时间

}

random_search = RandomizedSearchCV(svc, param_distributions, n_iter=10, cv=10,verbose=3)

random_search.fit(X_train,y_train)

#获取最佳参数

best_params=random_search.best_params_

print("随机搜索的最佳参数：",best_params)

best_model = random_search.best_estimator_

y_pred_train = best_model.predict(X_train)

y_pred_test=best_model.predict(X_test)

[accuracy_train,sensitivity_train,specificity_train,positive_predictive_values_train,negative_predictive_value_train,f1_train,kappa_train]=calculate_metrics_two(y_pred_train,y_train)

[accuracy_test,sensitivity_test,specificity_test,positive_predictive_values_test,negative_predictive_value_test,f1_test,kappa_test]=calculate_metrics_two(y_pred_test,y_test)、

**5.ANN**

# 初始化一个空列表来存储元组

tuples_list = []

# 使用循环从1到32生成元组，并将它们添加到列表中

for i in range(1, 33):

    if i < 32:

        tuples_list.append((i, i))

    else:

        tuples_list.append((i, i))  # 最后一个元组是(32, 32)

tuples_list

from scipy.stats import randint, uniform

#2层隐藏层

pipe=Pipeline([('MLP', MLPClassifier(activation='relu',solver='adam',alpha=0.1,learning_rate_init=0.001,tol=1e-4,n_iter_no_change=50))])#MLP中默认activation就是relu，solver就是‘adam’

param_grid={'MLP__hidden_layer_sizes':tuples_list,'MLP__max_iter':[500,1000,1500]}

grid=GridSearchCV(pipe,param_grid=param_grid,cv=10)

grid.fit(X_train,y_train)

#获取最佳参数

best_params=grid.best_params_

print("网格搜索的最佳参数：",best_params)

best_model = grid.best_estimator_

y_pred_train = best_model.predict(X_train)

y_pred_test=best_model.predict(X_test)

[accuracy_train,sensitivity_train,specificity_train,positive_predictive_values_train,negative_predictive_value_train,f1_train,kappa_train]=calculate_metrics_two(y_pred_train,y_train)

[accuracy_test,sensitivity_test,specificity_test,positive_predictive_values_test,negative_predictive_value_test,f1_test,kappa_test]=calculate_metrics_two(y_pred_test,y_test)

**6.Xgboost**

# 创建XGBoost分类器

xgb_clf = xgb.XGBClassifier(objective='binary:logistic', random_state=42)

# 定义超参数网格

param_grid = {

    'learning_rate': [0.01, 0.05, 0.1],               # 学习率

    'max_depth': [3, 6, 9],                            # 树的最大深度

    'n_estimators': [50, 100, 200],                    # 树的个数

    'subsample': [0.8, 0.9, 1.0],                      # 子样本比例

    'colsample_bytree': [0.8, 0.9, 1.0]                # 每棵树的列采样比例

}

grid=GridSearchCV(xgb_clf,param_grid=param_grid,cv=10)

grid.fit(X_train,y_train)

#获取最佳参数

best_params=grid.best_params_

print("随机搜索的最佳参数：",best_params)

best_model = grid.best_estimator_

y_pred_train = best_model.predict(X_train)

y_pred_test=best_model.predict(X_test)

[accuracy_train,sensitivity_train,specificity_train,positive_predictive_values_train,negative_predictive_value_train,f1_train,kappa_train]=calculate_metrics_two(y_pred_train,y_train)

[accuracy_test,sensitivity_test,specificity_test,positive_predictive_values_test,negative_predictive_value_test,f1_test,kappa_test]=calculate_metrics_two(y_pred_test,y_test)

**7.LDA**

# 创建LDA分类器

lda = LinearDiscriminantAnalysis()

# 定义超参数网格

param_grid = {

    'solver': ['svd', 'lsqr', 'eigen'],    # 选择求解器

    'shrinkage': [None, 'auto', 0.1, 0.5, 0.9]  # 收缩参数，仅在'lsqr'和'eigen'中使用

}

grid=GridSearchCV(lda,param_grid=param_grid,cv=10)

grid.fit(X_train,y_train)

#获取最佳参数

best_params=grid.best_params_

print("网格搜索的最佳参数：",best_params)

best_model = grid.best_estimator_

y_pred_train = best_model.predict(X_train)

y_pred_test=best_model.predict(X_test)

[accuracy_train,sensitivity_train,specificity_train,positive_predictive_values_train,negative_predictive_value_train,f1_train,kappa_train]=calculate_metrics_two(y_pred_train,y_train)

[accuracy_test,sensitivity_test,specificity_test,positive_predictive_values_test,negative_predictive_value_test,f1_test,kappa_test]=calculate_metrics_two(y_pred_test,y_test)

**8.Lightgbm**

# 创建LightGBM多分类分类器

lgbm_clf = lgb.LGBMClassifier(objective='binary', random_state=42)

# 定义超参数网格

param_distributions = {

    'learning_rate': [0.01, 0.05, 0.1],          # 学习率

    'num_leaves': [31, 40, 50],                  # 每棵树的最大叶子数

    'max_depth': [-1, 5, 10],                     # 树的最大深度

    'n_estimators': [100, 200, 300],             # 迭代次数（树的数量）

    'subsample': [0.8, 0.9, 1.0],                 # 子样本比例

    'colsample_bytree': [0.8, 0.9, 1.0],          # 每棵树的列采样比例

    'reg_alpha': [0, 0.1, 0.5],                   # L1正则化

    'reg_lambda': [0, 0.1, 0.5],                  # L2正则化

}

# 创建随机搜索对象

random_search = RandomizedSearchCV(lgbm_clf, param_distributions, n_iter=100, cv=10, random_state=42)

random_search.fit(X_train,y_train)

#获取最佳参数

best_params=random_search.best_params_

print("随机搜索的最佳参数：",best_params)

best_model = random_search.best_estimator_

y_pred_train = best_model.predict(X_train)

y_pred_test=best_model.predict(X_test)

[accuracy_train,sensitivity_train,specificity_train,positive_predictive_values_train,negative_predictive_value_train,f1_train,kappa_train]=calculate_metrics_two(y_pred_train,y_train)

[accuracy_test,sensitivity_test,specificity_test,positive_predictive_values_test,negative_predictive_value_test,f1_test,kappa_test]=calculate_metrics_two(y_pred_test,y_test)

**9.GBDT**

# 创建梯度提升树分类器对象

gbdt = GradientBoostingClassifier(random_state=42)

# 定义参数分布

param_dist = {

    "n_estimators": np.arange(50, 201, 10),

    "learning_rate": np.logspace(-3, 0, 4),

    "max_depth": [3, 4, 5, 6, 7],

    "min_samples_split": np.arange(2, 11),

    "min_samples_leaf": np.arange(1, 6)

}

# 创建随机搜索对象

random_search = RandomizedSearchCV(

    gbdt,

    param_distributions=param_dist,

    n_iter=100,

    cv=10,

    random_state=42,

    verbose=3

)

# 在训练集上进行网格搜索

random_search.fit(X_train, y_train)

#获取最佳参数

best_params=random_search.best_params_

print("网格搜索的最佳参数：",best_params)

best_model = random_search.best_estimator_

y_pred_train = best_model.predict(X_train_resampled)

y_pred_test=best_model.predict(X_test)

[accuracy_train,sensitivity_train,specificity_train,positive_predictive_values_train,negative_predictive_value_train,f1_train,kappa_train]=calculate_metrics_two(y_pred_train,y_train)

[accuracy_test,sensitivity_test,specificity_test,positive_predictive_values_test,negative_predictive_value_test,f1_test,kappa_test]=calculate_metrics_two(y_pred_test,y_test)
